# Endothelial Dysfunction and Metabolic Reprogramming in Cardiovascular Diseases

**DOI:** 10.31083/RCM51541

**Published:** 2026-09-17

**Authors:** Hongyu Chen, Yingliang Zhao, Chengxin Ge, Jianing Wang, Zhihua Wang, Hanzhang Wang, Shaopeng Cheng, Yilin Wang, Jie Yang, Dongjin Wang, Hoshun Chong, Chuiyu Kong

**Affiliations:** ^1^Department of Cardiac Surgery, Nanjing Drum Tower Hospital, Chinese Academy of Medical Sciences & Peking Union Medical College, 210008 Nanjing, Jiangsu, China; ^2^Department of Cardiac Surgery, Nanjing Drum Tower Hospital, Affiliated Hospital of Medical School, Nanjing University, 210008 Nanjing, Jiangsu, China; ^3^Institute of Cardiothoracic Vascular Disease, Nanjing University, 210008 Nanjing, Jiangsu, China; ^4^Department of Cardiothoracic Surgery, Nanjing Drum Tower Hospital Clinical College of Nanjing University of Chinese Medicine, 210008 Nanjing, Jiangsu, China

**Keywords:** endothelial cells, metabolic reprogramming, cardiovascular diseases

## Abstract

Endothelial cells regulate hemodynamics, inflammation, and vascular barrier integrity, thereby playing a central role in vascular homeostasis. Pathological conditions, including hypertension, hyperglycemia, and hyperlipidemia, induce metabolic reprogramming in these cells, altering the balance among glycolysis, fatty acid oxidation, and amino acid metabolism. This reprogramming drives endothelial dysfunction, characterized by reduced nitric oxide (NO) levels, increased inflammation and oxidative stress, and a weakened vascular barrier, thereby accelerating the progression of atherosclerosis, myocardial infarction, calcific aortic valve disease, and aortic aneurysms. Altered metabolites, such as lactate and acetyl-CoA, further exacerbate injury by modulating epigenetic changes and cell signaling. Endothelial metabolism has emerged as a promising therapeutic target for cardiovascular diseases. Natural compounds such as resveratrol, quercetin, and curcumin enhance NO production and reduce oxidative stress. Traditional Chinese medicine formulations (e.g., Qili Qiangxin and Tongxinluo capsules) help regulate glucose and lipid metabolism, thereby mitigating mitochondrial dysfunction and inflammation. Synthetic drugs, including metformin, sodium–glucose transporter 2 (SGLT2) inhibitors, glucagon-like peptide-1 receptor (GLP-1R) agonists, and experimental agents such as I-152, protect vessels by restoring metabolic homeostasis and endothelial function. Future research integrating multi-omics technologies and artificial intelligence is expected to enable real-time metabolic monitoring and precision interventions. Emerging delivery systems, such as exosome-based approaches and stem cell therapies, offer additional therapeutic potential. A deeper understanding of endothelial metabolic reprogramming will provide novel targets for preventing and treating cardiovascular diseases, thereby advancing precision medicine.

## 1. Introduction

Fatty acid carbon is essential for deoxyribonucleoside triphosphate (dNTP) synthesis in endothelial cells [1]. Despite the progress in the diagnosis and treatment of cardiovascular diseases (CVDs), they remain the primary causes of death worldwide, and the related morbidity and mortality are increasing [2]. Endothelial cells (ECs) are integral components of the vascular endothelium and play pivotal roles in maintaining vascular homeostasis, regulating hemodynamic parameters, and modulating inflammatory signaling cascades [3,4]. Under pathological conditions, ECs undergo active metabolic adaptation (e.g., regulating the dynamic reprogramming of essential metabolic pathways such as glycolysis, fatty acid β-oxidation (FAO), and amino acid metabolism) rather than suffering passive structural damage. This adaptive metabolic remodeling, referred to as metabolic reprogramming, is a pivotal mechanistic determinant in the development and progression of endothelial dysfunction [5,6].

Endothelial dysfunction primarily manifests as impaired vasodilation capacity, persistent proinflammatory and procoagulant states, and reduced nitric oxide (NO) bioavailability and is strongly correlated with the remodeling of the EC energy metabolism. Under normal physiological conditions, ECs rely on efficient glycolysis as a means of rapid energy supply to sustain stress responsiveness and maintain vascular-barrier homeostasis [7]. The increased glycolytic flux and decreased pentose phosphate levels caused by pathologies such as hypertension, hyperglycemia, and hyperlipidemia lead to the excessive production of nicotinamide adenine dinucleotide phosphate hydrogen (NADPH) oxidase (NOX) and reactive oxygen species (ROS), resulting in EC malfunction [8,9,10]. Disruptions in amino acid metabolism also contribute to endothelial injury [11]. The proliferation, migration, and inflammatory responses of ECs are regulated by the metabolic reprogramming of important amino acids (e.g., serine (Ser), glycine (Gly), and glutamine (Gln)), which affects carbon metabolism, epigenetic modification, and antioxidant synthesis [12,13,14]. Abnormal Gln catabolism can lead to the accumulation of metabolites such as α-ketoglutarate (α-KG), which, via epigenetic mechanisms, induces the persistent expression of proinflammatory genes, giving rise to the so-called metabolic memory effect [15]. Abnormalities in fatty acid metabolism exacerbate endothelial damage through multiple mechanisms including mitochondrial dysfunction, endoplasmic reticulum stress, and lipotoxic responses, with the excessive absorption of saturated fatty acids resulting in lipotoxic responses and mitochondrial dysfunction [16,17]. The proliferation of inflammatory mediators and activation of inflammasomes are facilitated by imbalances in polyunsaturated fatty acid metabolism [18]. Dysregulated lipid transport mediated by fatty acid–binding proteins leads to lipid accumulation, alters membrane fluidity, and inhibits endothelial NO synthase (eNOS), ultimately impairing vasodilation [19].

In summary, the metabolic reprogramming of ECs—including changes in the metabolism of glucose, fatty acids, and amino acids—plays a pivotal role in determining their function under pathological conditions. Based on the important role of ECs in the development of CVDs, this reprogramming is a promising target for CVD prevention and treatment. The present review summarizes recent progress in endothelial metabolic reprogramming and examines the potential implications of endothelial metabolites for clinical applications.

## 2. Metabolism in ECs

### 2.1 Carbohydrate Metabolism

Endothelial cells predominantly rely on glycolysis for energy production, generating up to approximately 85% of their ATP through this pathway. In rat coronary microvascular endothelial cells, approximately 98% of glucose is converted to lactate, whereas only about 0.04% is oxidized through the tricarboxylic acid (TCA) cycle [14]. Under normal circumstances, glucose is carried into the cytoplasm via glucose transporters 1 and 2 (GLUT1 and 2) and is then phosphorylated by hexokinase 2 (HK2) into glucose-6-phosphate (G6P). Next, fructose-6-phosphate (F6P), generated from G6P, is convert into 6-pax phosphate (F-2, 6-BP) by 6-phosphofructo-2-kinase/fructose-2,6-biphosphatase 3 (PFKFB3) [20,21,22]. As a key regulator of glycolysis, PFKFB3 catalyzes the conversion of fructose-6-phosphate (F6P) to fructose-2,6-bisphosphate (F2,6BP), a potent allosteric activator of phosphofructokinase 1 (PFK1). Activated PFK1 subsequently catalyzes the conversion of F6P to fructose-1,6-bisphosphate (F1,6BP), thereby promoting glycolytic flux. F1,6BP is then cleaved to generate glyceraldehyde-3-phosphate (G3P), which is further metabolized through a series of glycolytic reactions to form phosphoenolpyruvate (PEP). Pyruvate is largely (~98%) converted into lactate by lactate dehydrogenase, although a small proportion enters mitochondria to form acetyl-coenzyme A (acetyl-CoA) and participates in the TCA cycle [23]. Despite being directly exposed to oxygen-rich blood, ECs primarily rely on glycolysis, a metabolic pathway less energy-efficient than the TCA cycle. Glycolysis and the TCA cycle are very similar to each other in terms of their physiological functions for the following reasons. (I) By relying on oxygen-sparing glycolysis, ECs maximize oxygen diffusion to surrounding tissues while securing oxygen and nutrient delivery [24]. (II) In hypoxic tissue environments, glycolysis rapidly supplies energy to support EC migration and proliferation [25]. (III) Given that the leading cells guiding vessel growth during angiogenesis lack mitochondria, glycolysis is a more appropriate energy source for this highly dynamic structure [26]. (IV) Glycolysis generates less ROS, which helps alleviate oxidative damage to ECs [14]. (V) The final product of glycolysis—lactate—is a key signaling molecule promoting angiogenesis [27,28].

### 2.2 Glycolytic Bypass Pathway

Glycolytic bypass metabolism, which encompasses the pentose phosphate pathway (PPP) and the hexosamine biosynthesis pathway (HBP), plays a crucial role in ECs. In the process of producing NADPH to preserve vascular homeostasis [29], the PPP supplies precursors for nucleic acid synthesis, whereas the HBP is the primary metabolic pathway for protein glycosylation modification [30]. In the PPP, glucose-6-phosphate dehydrogenase catalyzes the conversion of G6P into NADPH and ribose-5-phosphate (R5P). NADPH—a crucial mediator in endothelial homeostasis—activates the deacetylase sirtuin 1 (SIRT1) to promote the deacetylation of eNOS and NO synthesis, thereby effectively regulating vascular relaxation [31]. Concurrently, NADPH can mitigate vascular oxidative stress by sustaining reduced glutathione (GSH) levels [32,33]. R5P is another metabolite used to induce nucleotide synthesis for EC proliferation and can be transformed into G6P under the action of transketolase at low glucose levels [34].

Under normal physiological conditions, the HBP integrates metabolic substrates—including F6P, Glu, and acetyl-CoA—to catalyze the production of uridine diphosphate-*N*-acetylglucosamine, which enables the rapid and reversible glycosylation of intracellular target proteins, modulating eNOS activity and the expression of cell proliferation–related genes to maintain endothelial function [35]. However, under pathological hyperglycemia conditions, the increased HBP flux triggers excessive O-linked β-N-acetylglucosamine (O-GlcNAc) modification to inhibit RAC-alpha serine/threonine-protein kinase (AKT)/eNOS phosphorylation, ultimately leading to eNOS dysfunction [36]. Abnormal advanced glycation end products (AGEs) activate inhibitor of kappa B kinase (IKK) complex formation via the AGEs/receptor for advanced glycation end products (RAGE) pathway, leading to the phosphorylation and degradation of nuclear factor of kappa light polypeptide gene enhancer in B-cells inhibitor (IKBα) and facilitating the nuclear translocation of nuclear factor kappa-B (NF-κB) to enhance its transcriptional activity and disrupt normal negative feedback regulation. Ultimately, the continuous release of inflammatory mediators such as Interleukin-1 (IL-1) and Interleukin-6 (IL-6) worsens vascular inflammation [37].

Thus, glucose metabolism not only furnishes the majority of energy through glycolysis but also provides substrates for the synthesis of deoxyribonucleic acid and NADPH (an essential reducing agent) via the PPP and HBP, maintaining homeostasis in ECs.

### 2.3 Fatty Acid Metabolism

FAO contributes to ~5% of the total energy supply in ECs, generating diverse metabolic intermediates rather than serving as a major bioenergetic source [17]. In a proliferative state, ECs upregulate the expression of fatty acid transporters 3 and 4 (FATP3 and 4) in response to vascular endothelial growth factor B, enhancing the uptake of long-chain fatty acids (LCFAs). Once internalized, LCFAs bind to fatty acid binding proteins 4 and 5 (FABP4 and 5) within the cytoplasm and are subsequently trafficked to mitochondria and imported into the mitochondrial matrix via the rate-limiting outer membrane enzyme carnitine palmitoyltransferase 1a (CPT1A) [38]. Within the matrix, fatty acids undergo FAO to produce acetyl-CoA, which is not only involved in the TCA cycle for energy supply but also provides carbon skeleton precursors for the *de novo* synthesis of purine/pyrimidine nucleotides [1,6]. The knockout of *CPT1A* notably suppresses pyrimidine nucleotide biosynthesis [1].

Acetyl-CoA carboxylase (ACC) is the main rate-limiting enzyme in the metabolism of fatty acids, comprising two subunits (ACC1 and ACC2). ACC1 is found in the cytoplasm, where it facilitates the carboxylation of acetyl-CoA to produce malonyl-CoA, which is subsequently transformed into fatty acids by fatty acid synthase (FASN) [39]. ACC2 acts as a negative regulator of *CPT1A* activity and is expressed in the outer mitochondrial membrane [39]. Consequently, the metabolic intermediation of malonyl-CoA, which is a major metabolic intermediate, has a negative effect on ACC2-mediated FAO and a positive effect on the synthesis of fatty acids, which controls the dynamic regulation of the metabolic flux of fatty acids in ECs [40].

In summary, although fatty acid metabolism is not the principal source of energy in ECs, it plays a crucial regulatory role in maintaining cellular proliferation, metabolic equilibrium, and vascular homeostasis.

### 2.4 Amino Acid Metabolism

Amino acid metabolism is essential for EC proliferation and migration. Gln—the most common nonessential amino acid in plasma (~0.65 mM)—is a major nitrogen donor and metabolic substrate. Beyond supplying carbon skeletons and nitrogen for macromolecular biosynthesis, Gln sustains a broad spectrum of metabolic and signaling pathways essential for endothelial homeostasis [41]. A substantial proportion of extracellular Gln is taken up through the sodium-dependent neutral amino acid transporter solute carrier family 1 member 5 (SLC1A5/ASCT2). Intracellular Gln can subsequently serve as an exchange substrate for the L-type amino acid transporter solute carrier family 7 member 5 (SLC7A5/LAT1), thereby facilitating the uptake of extracellular essential amino acids [42]. This process is crucial for maintaining the intracellular–extracellular flux of Gln, which is essential for metabolic homeostasis and signaling regulation in ECs. Under physiological conditions, Gln undergoes deamidation catalyzed by glutaminase isoforms (GLS1 and GLS2) to generate glutamate (Glu), thereby furnishing cells with essential nitrogen donors and carbon skeletons for biosynthetic and energetic demands. Subsequently, Glu is oxidatively deaminated by glutamate dehydrogenase to yield α-KG, which enters the TCA cycle to participate in mitochondrial bioenergetics, while Gln-derived intermediates concurrently contribute to GSH biosynthesis, thereby preserving redox homeostasis and supporting nucleotide anabolism [13,43]. As a central intermediate in Gln metabolism, Glu exerts multiple regulatory functions within ECs. On the one hand, Glu can be catalytically converted into Gln by glutamine synthetase and then exported via SLC7A5 to replenish extracellular Gln pools in accordance with systemic requirements. On the other hand, along with Gly and cysteine (Cys), Glu participates in the biosynthesis of GSH, a crucial antioxidant metabolite protecting ECs from oxidative stress [44,45]. Glutamate can be reversibly converted to α-ketoglutarate by glutamate dehydrogenase (GDH), thereby linking glutamate metabolism to the TCA cycle. Glutamate also contributes to ornithine and polyamine metabolism through separate enzymatic pathways [46]. PAs are crucial metabolic regulators of EC proliferation and angiogenesis, facilitating cell cycle progression by stabilizing nucleic acid structures and enhancing translational efficiency while modulating eNOS activity to fine-tune NO bioavailability and thereby orchestrating the endothelial vasodilatory function and vascular homeostasis [47]. Apart from the Glu-dependent pathway, arginine (Arg) metabolism contributes to the generation of PAs and urea via arginase (ARG), which competes with eNOS for the common substrate. The pharmacological inhibition of ARG or exogenous Arg supplementation can restore the function of eNOS and mitigate oxidative stress–induced endothelial injury, thereby preserving vascular integrity and homeostasis [48]. Serine is converted to glycine by serine hydroxymethyltransferase (SHMT), which simultaneously transfers a one-carbon unit to tetrahydrofolate (THF), thereby linking serine/glycine metabolism to the one-carbon cycle. Gly serves as an indispensable substrate for heme biosynthesis, while THF functions as a crucial precursor for purine synthesis. Along with methionine, Gly is involved in the generation of S-adenosylmethionine (SAM), a universal methyl donor that mediates epigenetic modifications such as histone methylation [49].

Collectively, amino acids such as Gln, Arg, and Ser orchestrate the function of ECs through an intricate metabolic network, not only furnishing essential biosynthetic precursors, including nucleotides and PAS, to sustain proliferative capacity but also maintaining redox homeostasis via GSH synthesis and modulating epigenetic regulation through one-carbon metabolism, thereby coordinately governing the survival, migration, and angiogenic activity of ECs.

### 2.5 Interconnections Among Metabolic Pathways

Acetyl-CoA serves as a pivotal hub in glucose, lipid, and amino acid metabolism, occupying a central position within cellular metabolic networks. During glucose metabolism, pyruvate produced via glycolysis is converted into acetyl-CoA, which enters the TCA cycle [23]. In lipid metabolism, acetyl-CoA serves as a precursor for fatty acid synthesis, while its carboxylation product, malonyl-CoA, regulates the synthesis and β-oxidation of fatty acids [50]. In amino acid metabolism, branched-chain amino acids (such as leucine, isoleucine, and valine) and aromatic amino acids (such as phenylalanine and tyrosine) can be converted into acetyl-CoA through different pathways, participating in energy metabolism. This convergence of multiple metabolic pathways allows cells to flexibly regulate the metabolic flux according to physiological demands and nutritional status and thus ensure homeostasis (Fig. 1).

**Fig. 1.**
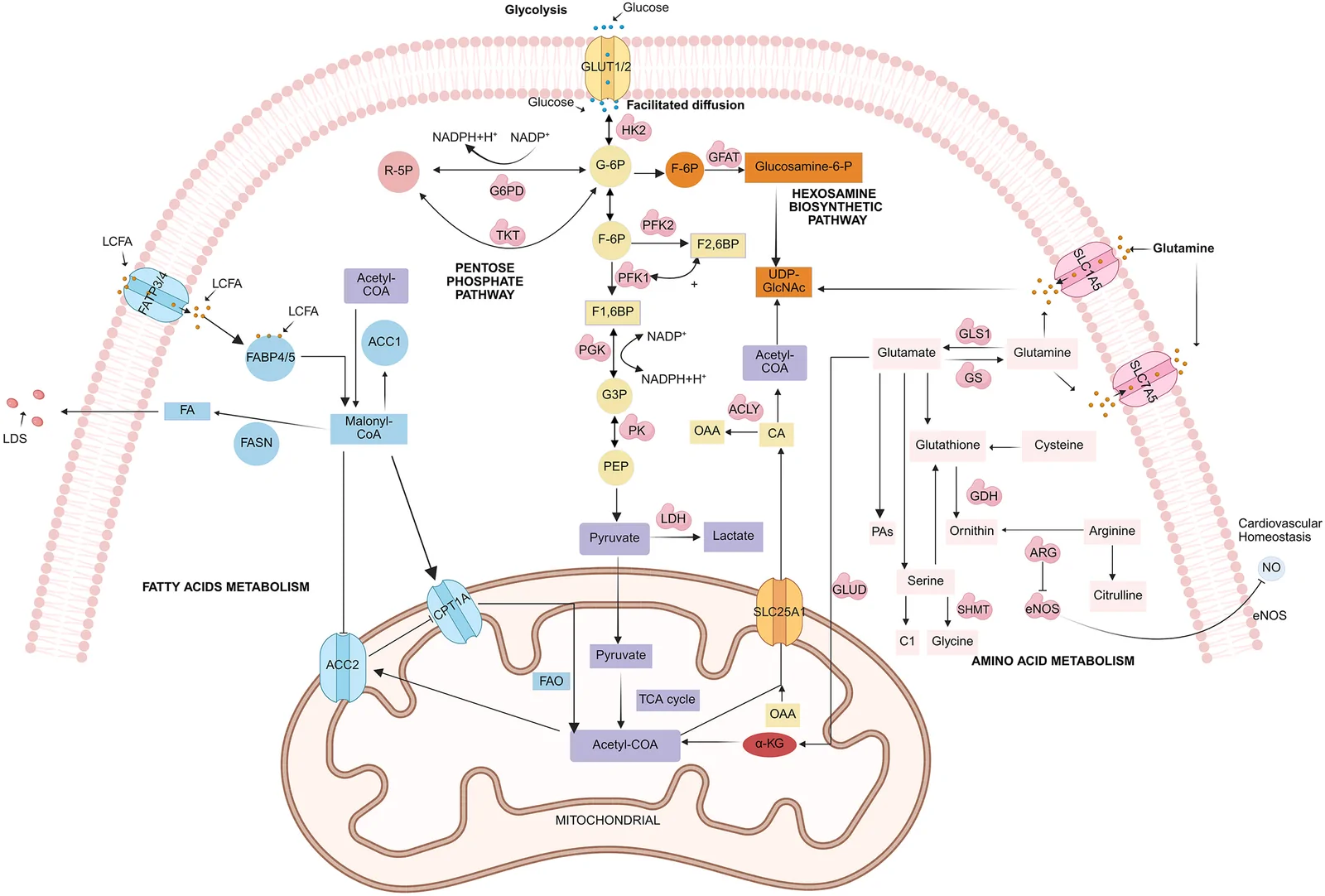
**Integrated metabolic network of endothelial cells linking the metabolism of glucose, fatty acids, and amino acids**. Schematic metabolic pathways of glucose, fatty acids, and amino acids regulating endothelial energy homeostasis and vascular function. In the schematic, arrows indicate the direction of metabolic conversion or regulatory flow, whereas T-shaped lines indicate inhibitory effects. Different colors are used to distinguish the major metabolic modules: yellow/orange indicates glucose metabolism and glycolysis-related intermediates, blue indicates fatty acid metabolism, pink indicates amino acid metabolism, and purple indicates mitochondrial/TCA cycle-related metabolites and pathways. ACC, Acetyl-CoA carboxylase; LCFA, long-chain fatty acid; FA, fatty acid; FASN, fatty acid synthase; LDS, lipid droplets; OAA, oxaloacetate; PGK, phosphoglycerate kinase; GFAT, glutamine:fructose-6-phosphate amidotransferase; PEP, phosphoenolpyruvate; FAO, fatty acid β-oxidation; GS, glutamine synthetase; ARG, arginase; eNOS, endothelial NO synthase. Created in BioRender.

## 3. Endothelial Dysfunction and Metabolic Reprogramming in CVDs

Endothelial metabolic reprogramming provides a mechanistic framework linking pathological stimuli to vascular injury across different cardiovascular diseases. In response to hyperglycemia, dyslipidemia, hypoxia, inflammatory activation, and abnormal shear stress, ECs undergo coordinated alterations in glucose, lipid/fatty acid, and amino acid metabolism. These metabolic disturbances converge on several common functional consequences, including reduced NO bioavailability, eNOS uncoupling, ROS accumulation, mitochondrial dysfunction, inflammatory activation, barrier disruption, and maladaptive intercellular communication (Fig. 2). In parallel with these pathway-level alterations, multiple metabolites and metabolic intermediates have been identified as disease-associated biomarkers or functional mediators of endothelial dysfunction and cardiovascular diseases. These metabolites reflect disturbances in glycolysis, redox metabolism, amino acid metabolism, lipid metabolism, and endothelial NO signaling (Table 1, Ref. [13,33,35,36,37,43,45,51,52,53,54,55,56,57,58,59,60,61,62,63,64,65,66,67,68,69,70,71,72,73,74,75,76,77,78,79,80,81,82,83,84,85,86,87,88,89,90]). Collectively, these shared endothelial abnormalities contribute to the development and progression of AS, MI, CAVD, AA/AAD, and heart failure, although each disease context is characterized by distinct upstream triggers and dominant downstream pathways.

**Fig. 2.**
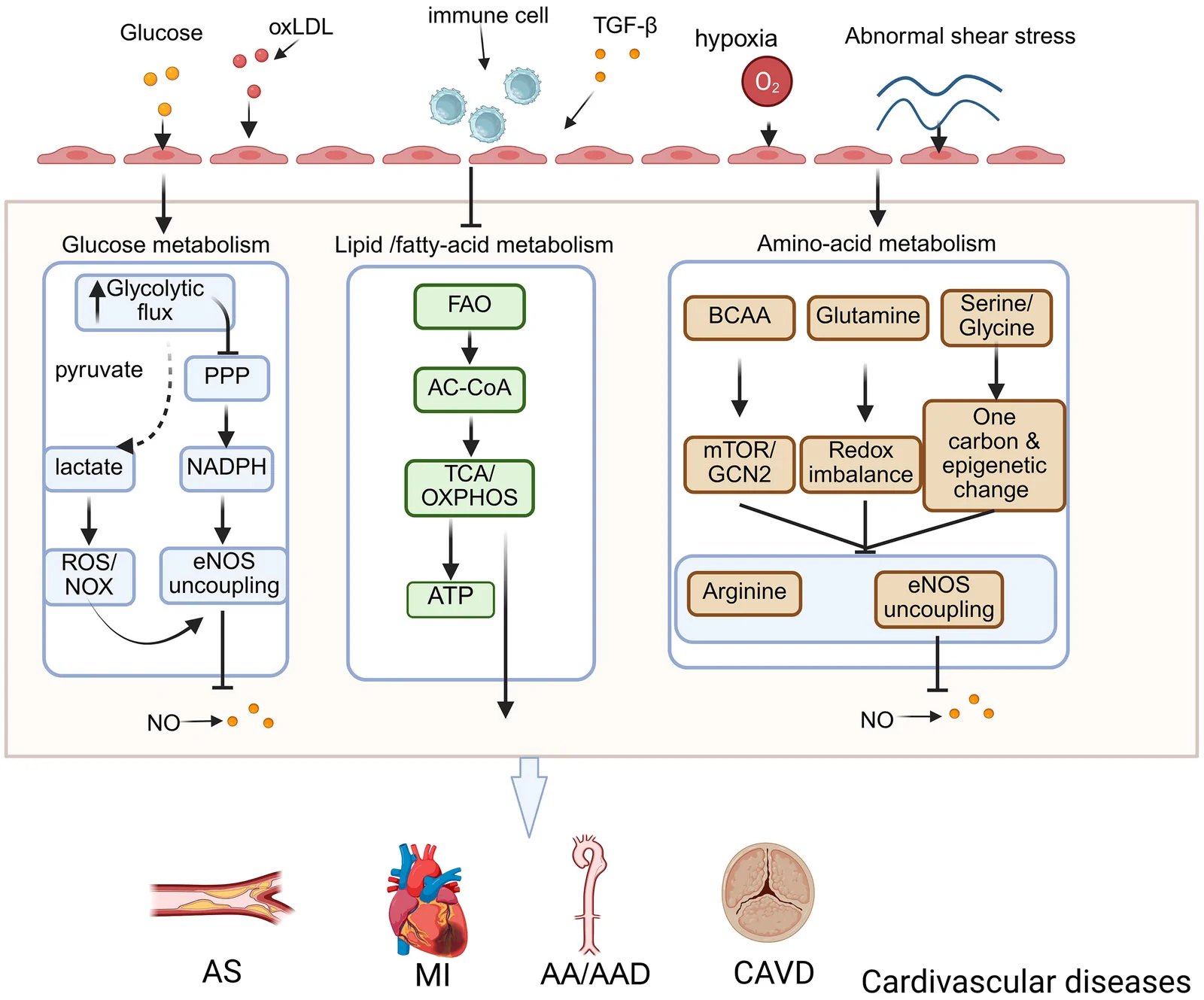
**Endothelial Dysfunction and Metabolic Reprogramming in CVDs**. Pathological stimuli, including hyperglycemia, oxLDL, inflammatory signals, TGF-β, hypoxia, and abnormal shear stress, induce endothelial metabolic reprogramming involving glucose, lipid/fatty-acid, and amino-acid metabolism. These metabolic alterations impair NO bioavailability, promote oxidative stress, mitochondrial dysfunction, eNOS uncoupling, and epigenetic remodeling, thereby contributing to the progression of AS, MI, AA/AAD, and CAVD. In the schematic, solid arrows indicate the direction of metabolic flow or regulatory effects, dashed arrows indicate metabolic conversion or branching flux, and T-shaped lines indicate inhibitory effects. The large outlined arrow indicates the overall contribution of endothelial metabolic reprogramming to cardiovascular diseases. CVDs, cardiovascular diseases; oxLDL, oxidized low-density lipoprotein; TGF-β, transforming growth factor-beta; AS, atherosclerosis; MI, myocardial infarction; AA/AAD, aortic aneurysms/aortic dissections. Created in BioRender.

**Table 1.** **Endothelial dysfunction and metabolic reprogramming in CVDs**.

Full metabolite name	Abbreviation	Change in disease	References
Lactate	Lactate	↑ AS; ↑ MI; ↑ AA/AAD	[75,81,84,85]
Acetyl-coenzyme A	Acetyl-CoA	↑ CAVD; ↑ AA/AAD	[80,89]
Nicotinamide adenine dinucleotide phosphate	NADPH	↓ AS; ↓ MI	[72,85]
Ribose-5-phosphate	R5P	↓ MI	[85]
Tetrahydrobiopterin	BH4	↓ CAVD; ↓ MI	[78,86]
Dihydrobiopterin	BH2	↑ MI	[86]
Nitric oxide	NO	↓ CAVD; ↓ AS; ↓ MI	[51,52,53,58,63,66,67,73,78,86]
Glutamine-to-glutamate ratio	GGR/Gln/Glu ratio	↓ AS	[55,56,57,83]
Glutamine	Gln	↓ AS; ↓ ischemic vascular injury; ↑ CAVD	[13,55,56,57,80,83]
Glutamate	Glu	↑ AS	[55,56,57,83]
L-Arginine	L-Arginine/Arginine	↓ AS	[58,73]
Branched-chain amino acids	BCAAs	↑ AS; ↑ MI	[59,60,61,62,87,88]
Homocysteine	Hcy	↑ AS; ↑ AA/AAD	[63,64,65,77]
Asymmetric dimethylarginine	ADMA	↑ AS; ↑ CKD; ↑ Cushing syndrome	[63,66,67]
Long-chain fatty acids	LCFAs	↑ AS	[68]
Oxidized low-density lipoprotein	oxLDL	↑ CAVD; ↑ AS	[54,69,70,71,72,74]
Lipoprotein(a)	Lp(a)	↑ CAVD	[54]
Lysophosphatidic acid	LPA	↑ CAVD	[54]
Uric acid	UA	↑ CAVD	[79]
Advanced glycation end products	AGEs	↑ diabetic vascular complications	[37]
O-linked β-N-acetylglucosamine modification	O-GlcNAc	↑ diabetic vascular complications	[35,36]
Glutathione	GSH	↓ endothelial oxidative stress–associated CVDs	[33,43,45,82,90]
Adenosine triphosphate	ATP	↓ AA/AAD	[76]

The table summarizes disease-associated metabolic biomarkers described in our manuscript, including their full names, abbreviations, reported changes in disease contexts, and corresponding references. Upward and downward arrows indicate increased and decreased levels, respectively. AS, atherosclerosis; MI, myocardial infarction; AA/AAD, aortic aneurysms/aortic dissections; CAVD, calcific aortic valve disease; CKD, chronic kidney disease.

### 3.1 Calcific Aortic Valve Diseases (CAVD)

The aortic valve comprises valvular endothelial cells (VECs), valvular interstitial cells (VICs), and the extracellular matrix (Fig. 3). VECs line the valve surface and function as microenvironment sensors responding to mechanical and biochemical stimuli. Prolonged pathological stimulation induces inflammatory responses and oxidative stress, thus leading to cellular metabolic reprogramming that contributes to the progression of CAVD [91]. eNOS is a crucial enzyme for preserving endothelial function, with its dysfunction markedly decreasing NO bioavailability and thus resulting in CAVD. eNOS-global-knockout mice were shown to feature a higher incidence of aortic valve stenosis and bicuspid aortic valve malformation than wild-type mice, which was ascribed to the notably reduced NO production in the former group [92]. When tetrahydrobiopterin (BH4)—a critical predictor of the eNOS biofunction—and its biosynthetic enzymes GTP cyclohydrolase I (GCH1) and dihydrofolate reductase (DHFR) were knocked out in murine ECs, the progression of aortic valve calcification in *Apoe*^-/-^ mice accelerated [78]. When these mice were treated with folic acid or BH4, aortic valve calcification was considerably delayed. *In vitro* studies have demonstrated that the exogenous supplementation of NO in osteogenic differentiation media for VICs substantially mitigates the calcification phenotype [51]. Abnormal uric acid metabolism may also drive aortic calcification and promote the osteogenic differentiation of aortic VECs by inhibiting hypoxia-inducible factor 1α (HIF-1α) and thereby disrupting the endothelial barrier function [79]. Shear stress is a key regulator of eNOS activity and plays a crucial role in the development of CAVD [51]. Laminar shear stress increases the expression of eNOS in VECs and boosts cyclic guanosine monophosphate (cGMP) signaling, which subsequently suppresses the osteogenic differentiation of VICs [52]. Conversely, low shear stress or oscillatory shear stress decreases the expression of eNOS, reduces the availability of NO, and induces oxidative stress, thus promoting phenotypic changes in VECs and accelerating the progression of CAVD [53]. Under normal physiological conditions, VICs remain in a quiescent state maintained by paracrine signaling from adjacent VECs; however, upon VIC injury, VECs can undergo phenotypic conversion into endothelial-derived VICs, thereby contributing to valve repair [93]. Under conditions of ongoing injury, VICs at the lesion site facilitate the buildup of oxidized low-density lipoprotein (oxLDL) and lipoprotein a, which promotes the infiltration of proinflammatory macrophages and monocytes. This process indirectly activates ENPP2 (a gene encoding autotaxin) and lysophosphatidic acid signaling, ultimately steering ECs toward osteogenic differentiation [54]. The pathophysiology of CAVD critically depends on these processes [94]. During the abovementioned process, the sustained activation of the transforming growth factor-beta (TGF-β)- SMAD signaling pathway not only increases endothelial-to-mesenchymal transition (EndMT) levels but also inhibits the function of the integrin 3–linked kinase (ILK)/eNOS/NO axis, which drives valve calcification by upregulating runt-associated transcription factor 2 (RUNX2), an osteogenic transcription factor [92].

**Fig. 3.**
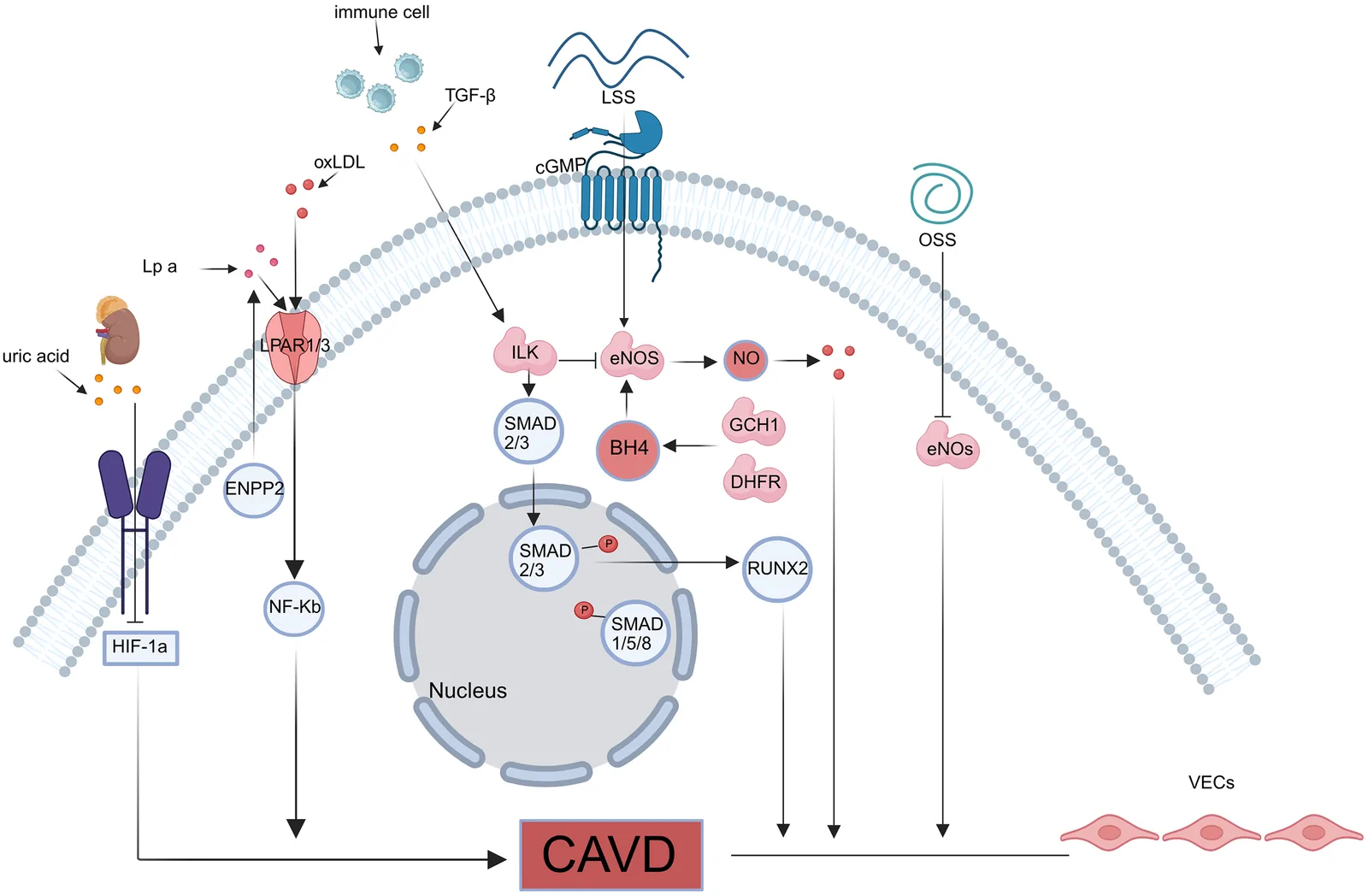
**Schematic illustration of signaling pathways associated with valvular endothelial cell dysfunction in calcific aortic valve disease (CAVD)**. Pathological and mechanical stimuli, including uric acid, lipoprotein(a) [Lp(a)]/oxidized low-density lipoprotein (oxLDL), TGF-β, laminar shear stress (LSS), and oscillatory shear stress (OSS), regulate endothelial signaling through HIF-1α, NF-κB, ILK/SMAD2/3, and eNOS/NO-related pathways. BH4 metabolism, regulated by GCH1 and DHFR, contributes to eNOS function and NO bioavailability, while SMAD signaling and RUNX2 are associated with osteogenic responses. Together, these alterations contribute to valvular endothelial cell dysfunction and CAVD progression. Created in BioRender. Created in BioRender.

Few studies have focused on the regulation of calcification by metabolic reprogramming in VECs, with most works focusing on the metabolic changes triggered by VEC–VIC interactions. High shear stress or treatment with the piezo1 activator Yoda1 may increase the amount of piezo proteins in VECs and thus cause Ca^2+^ inflow and encourage Yes-associated protein (YAP) translocation to the nucleus. This effect stimulates GLS1-mediated Gln catabolism, generating acetyl-CoA, which enhances histone H3 acetylation at the* RUNX2* promoter region, thereby exacerbating osteogenic lesions in VICs [80].

In summary, the core mechanism of CAVD development involves pathological EndMT and dysfunction in VECs. Through the intersection of multiple signaling pathways, changes in VECs promote the pathological osteogenic transformation of VICs, with key roles played by weakened eNOS/NO signaling, abnormal mechanosensing, and the activation of several pathways.

### 3.2 Atherosclerosis (AS)

As an important barrier for maintaining vascular homeostasis, ECs can experience functional dysregulation because of metabolic reprogramming, which is a core characteristic of the onset and progression of early-stage AS [95] (Fig. 4). In this context, glycolysis—the main bioenergetic pathway in ECs—undermines vascular barrier integrity when dysregulated, initiates persistent inflammatory activation, and intensifies oxidative stress, thus converting intracellular metabolic imbalance into progressive vascular injury [8]. The excessive activation of PFKFB3—the key rate-limiting enzyme—not only amplifies glycolytic flux and induces lactate accumulation but also phosphorylates the inhibitor of NF-κB (IκB) at Ser536 to activate IKK complex formation, leading to IκB degradation and subsequent NF-κB nuclear translocation. These processes initiate the transcription of proinflammatory mediators and accelerate the pathophysiological progression of AS [81]. The pharmacological or genetic inhibition of PFKFB3 attenuates endothelial injury, thereby enhancing atherosclerotic plaque stability and decelerating the progression of coronary AS through the preservation of endothelial integrity and suppression of inflammatory activation [96]. Altered amino acid metabolism in ECs plays an important role in the onset and advancement of vascular diseases, mainly by hindering NO production and disrupting NADPH balance, both of which are crucial for preserving endothelial function and vascular integrity [82]. Gln-to-Glu ratio (GGR) has been associated with AS and plaque progression, while emerging evidence suggests that Gln/Glu metabolism may also participate in vascular and valvular calcification [55,56,83]. GLS2 might influence plasma GGR to adjust the metabolic interactions between ECs and perivascular cells, as well as control aortic structural remodeling. In contrast, the decreased expression of GLS2 accelerates plaque progression and arterial wall stiffening, thereby facilitating the progression of atherosclerotic pathology [57]. Moreover, urea cycle disruption has been linked to the aggravation of AS, as the inhibition of sirtuin 3 leads to the inactivation of argininosuccinate synthase 1, thereby hindering eNOS-mediated NO synthesis, increasing ROS accumulation, and decreasing L-arginine availability [58]. The risk factors for AS include hyperhomocysteinemia and branched-chain amino acids (BCAAs). BCAA dysfunction promotes lipid accumulation in macrophages, drives foam cell formation, and impairs endothelial function [59]. Rats with insulin resistance are known to exhibit elevated plasma leucine levels, which are associated with impaired endothelium-dependent vasodilation through the activation of the mTOR/NF-κB pathway and lead to reduced NO availability and increased oxidative stress [60]. Although the function of valine in AS has not been deeply studied, its metabolite, secreted by skeletal muscle—3-hydroxyisobutyrate—enhances fatty acid uptake in ECs, causing intramuscular lipid deposition and insulin resistance [61]. The plasma levels of BCAAs and their metabolic intermediates are negatively correlated with cardiac function and positively correlated with insulin resistance, possibly because of the negative correlation of these levels with the activity of catabolic enzymes [62]. Homocysteine (Hcy), which serves as an intermediate in the folate metabolic pathway, is typically regulated by SAM and acts as a methyl donor. After being metabolized to S-adenosylhomocysteine, SAM is hydrolyzed to Hcy, which can be remethylated to methionine via methionine synthase or irreversibly converted to Cys through the cystathionine β-synthase pathway, ultimately contributing to GSH synthesis. Hcy accumulation reflects impaired folate cycling, resulting in reduced methylation, increased ROS levels, and endoplasmic reticulum stress, all of which reduce endothelial function [63,64,65]. In pathological conditions such as CKD and Cushing syndrome, the accumulation of asymmetric dimethylarginine inhibits eNOS and thereby promotes AS development [66,67].

**Fig. 4.**
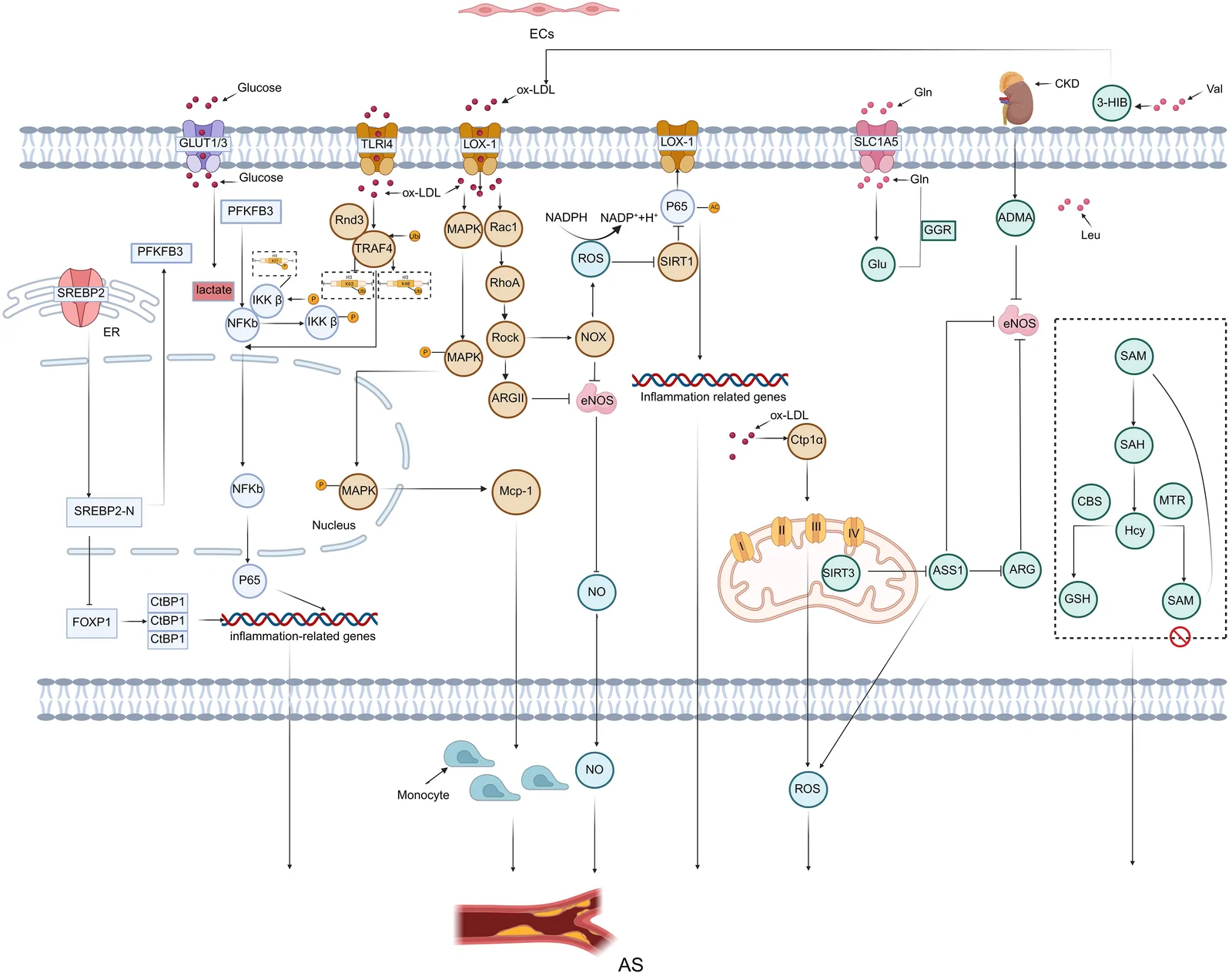
**Mechanistic illustration of metabolic reprogramming–induced endothelial dysfunction contributing to atherogenesis**. Augmented glycolytic flux driven by PFKFB3 activation following glucose uptake via GLUT1/3 promotes lactate accumulation, thereby enhancing NF-κB signaling and the transcription of proinflammatory mediators. Concurrently, oxidized low-density lipoprotein (oxLDL) binding to toll-like receptor 4 (TLR4) and lectin-like oxidized LDL receptor 1 (LOX-1) activates mitogen-activated protein kinase (MAPK), RhoA, and Rho-associated coiled-coil containing protein kinase (ROCK) signaling, promoting NADPH oxidase–dependent reactive oxygen species (ROS) generation, eNOS uncoupling, and nitric oxide (NO) depletion. Perturbations in amino acid metabolism, characterized by a reduced Gln/Glu ratio and elevated asymmetric dimethylarginine levels, further disrupt redox homeostasis and endothelial integrity. In addition, SIRT3 downregulation impairs argininosuccinate synthase 1 activity, reduces L-arginine bioavailability, and promotes mitochondrial oxidative stress, thereby enhancing endothelial inflammation, leukocyte adhesion, and atherosclerotic plaque progression. Created in BioRender.

Dysregulated lipid metabolism is also a critical metabolic basis for endothelial dysfunction. Excess lipids exert lipotoxic effects and induce changes in energy metabolism. Dysregulated lipid metabolism, along with glucose and amino acid metabolism, accelerates disease progression. Elevated plasma levels of long-chain fatty acids can inhibit EC proliferation and induce vascular inflammation by activating the IKK/NF-κB pathway [68]. An important stage of AS development is the increased internalization of oxLDL [69]. The main oxLDL transporter (LOX-1) activates the mitogen-activated protein kinase (MAPK)- monocyte chemoattractant protein-1 (MCP-1) pathway upon binding to oxLDL, thus increasing NOX levels and enhancing ROS production [70,71], stimulating the infiltration of monocytes, and interacting with matrix metalloproteinase-14 (MMP14) to activate Rac1/RhoA signaling. Sustained NOX activation consumes NADPH and promotes ROS accumulation, thereby exacerbating endothelial oxidative stress [72]. In parallel, reduced SIRT1 activity impairs the deacetylation of NF-κB p65, resulting in persistent NF-κB activation, inflammatory signaling, and apoptosis [97]. At the same time, elevated ARG activity decreases L-arginine levels, impairing eNOS activity and reducing NO production [73]. The activation of the ROS/NF-κB pathway further upregulates LOX-1, establishing a vicious cycle [74]. As a result, poor prognosis is typically linked to high LOX-1 expression. Excess lipid accumulation also upregulates fatty acid oxidation genes such as* CPT1A*, and hyperactive FAO may overload the mitochondrial electron transport chain, increasing ROS leakage [98].

The metabolic reprogramming of ECs is one of the main processes involved in the onset and progression of AS. The abnormal levels of metabolites in amino acid metabolism, glucose metabolism and lipid metabolism also disturb the endothelium and lead to oxidative stress, inflammatory reactions, and decreased NO signaling, which in turn increases the formation and progression of plaque. Important endothelial metabolites hold great promise for the prevention and treatment of AS.

### 3.3 Myocardial Infarction (MI)

Owing to their glycolysis-based energy supply, ECs exhibit a strong tolerance to hypoxia via aerobic metabolism during MI, featuring a survival rate markedly exceeding that of cardiomyocytes (Fig. 5). However, prolonged ischemia resulting from persistent coronary occlusion can still lead to endothelial energy depletion (which causes microvascular dysfunction or even cell death), accompanied by metabolic reprogramming and microcirculatory collapse [99]. Microvascular obstruction is not only a pathological consequence of MI but also a key factor that exacerbates myocardial necrosis, expands infarct size, and worsens prognosis by limiting perfusion and enhancing inflammation and oxidative stress [100,101].

**Fig. 5.**
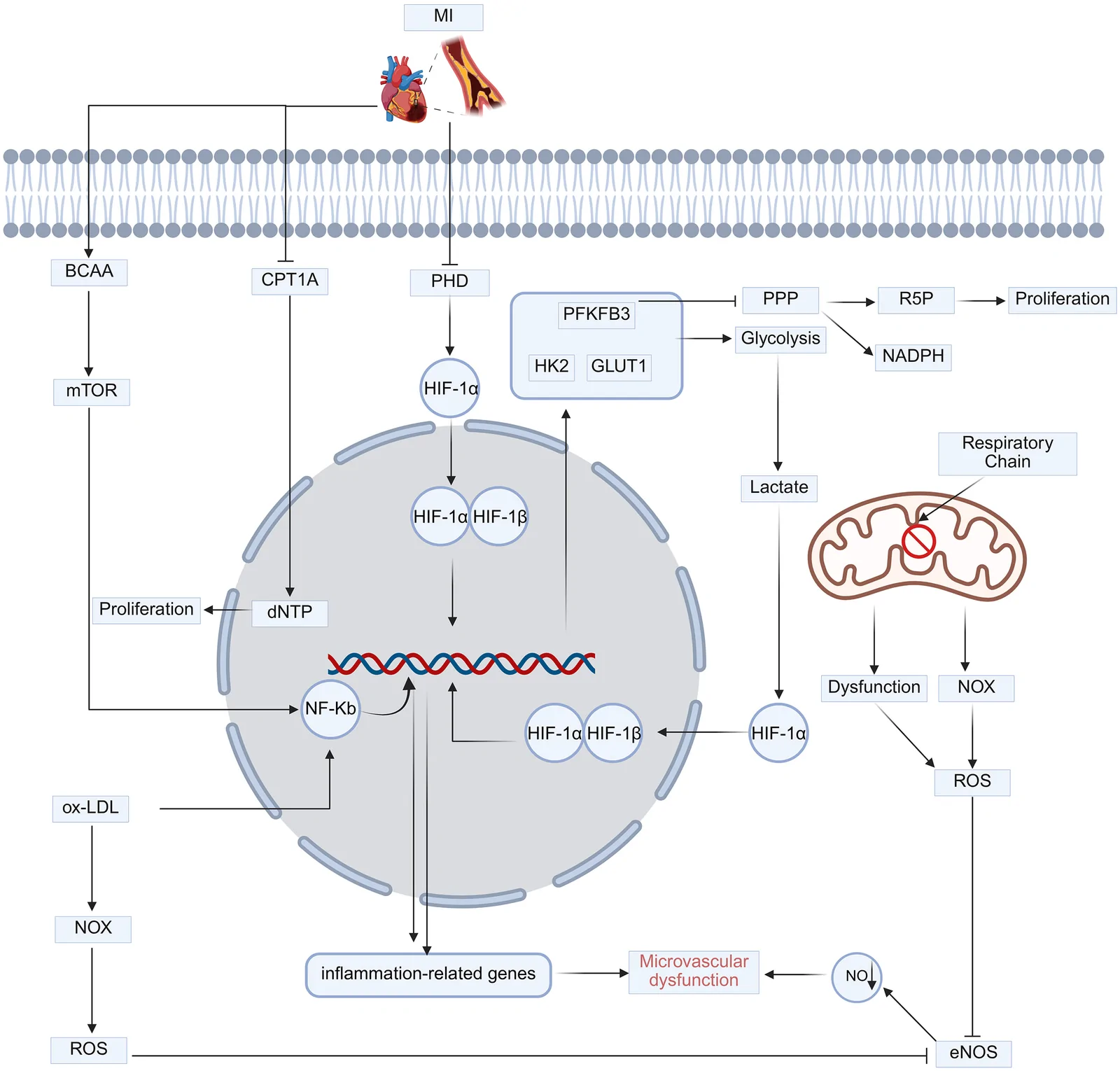
**Metabolic reprogramming and endothelial dysfunction in myocardial infarction**. Myocardial infarction (MI) induces multiple metabolic and signaling alterations in endothelial cells. Increased branched-chain amino acid (BCAA) signaling activates mTOR and contributes to NF-κB–mediated inflammatory responses. Alterations in CPT1A-dependent fatty acid metabolism are associated with changes in dNTP availability and endothelial proliferative capacity. Inhibition of prolyl hydroxylase domain protein (PHD) promotes HIF-1α stabilization and HIF-1α/HIF-1β–dependent transcriptional responses, including the upregulation of glycolytic regulators such as PFKFB3, HK2, and GLUT1. Enhanced glycolysis promotes lactate accumulation, while the pentose phosphate pathway (PPP) generates ribose-5-phosphate (R5P) and NADPH, thereby contributing to nucleotide synthesis, redox regulation, and endothelial proliferation. Mitochondrial respiratory-chain dysfunction is associated with increased reactive oxygen species (ROS) production and endothelial dysfunction. In parallel, oxLDL-induced oxidative stress and NOX activation, together with altered eNOS/NO signaling and NF-κB–dependent inflammation-related gene expression, contribute to microvascular dysfunction following MI. Created in BioRender.

Under hypoxic conditions, the inhibition of prolyl hydroxylase hinders the hydroxylation of HIF-1α, allowing it to dimerize with hypoxia-inducible factor 1-beta and translocate into the nucleus to upregulate glycolysis-related genes such as GLUT1, HK2, and PFKFB3, while accumulated lactate further amplifies this response by activating HIF-1α in a positive feedback loop [84]. PFKFB3-driven glycolytic enhancement may divert glucose flux away from the PPP, thereby reducing NADPH and R5P production and impairing antioxidant capacity. Although mitochondrial NADPH can partially compensate for cytosolic NADPH through the isocitrate/α-KG shuttle, this may disrupt iron-sulfur cluster biogenesis and exacerbate mitochondrial dysfunction [85]. FAO and amino acid metabolism are further disrupted by mitochondrial damage. Under the conditions of intense oxidative stress and inflammation, the inhibition of *CPT1A* impairs deoxynucleotide synthesis, thereby limiting EC proliferation [1]. Impaired lipolysis leads to intracellular lipid accumulation, which activates the *NF-κB* pathway and promotes the release of inflammatory cytokines, while oxLDL stimulation further increases ROS production [102,103]. BH4, a crucial cofactor for eNOS, can be oxidized by ROS to dihydrobiopterin, which leads to eNOS uncoupling, increases superoxide production, and aggravates vascular damage [86].

Research on amino acid metabolism in ECs during MI is limited. The dysfunction of BCAA catabolic enzymes following MI was suggested to cause excessive BCAA accumulation, *mTOR* signaling activation, and subsequent cardiac dysfunction [87,88]. In summary, endothelial metabolic reprogramming constitutes a central mechanism underlying microcirculatory dysfunction in MI. Abnormal glycolysis, along with dysregulated lipid and amino acid metabolism, promotes oxidative stress and inflammation, resulting in eNOS dysfunction and microvascular collapse and ultimately exacerbating MI.

### 3.4 Aortic Aneurysms and Aortic Dissections (AA/AAD)

Along with smooth muscle cells and macrophages, the metabolic abnormalities and functional impairments of ECs play a crucial role in the development and progression of aortic aneurysms and aortic dissections (Fig. 6). The specific knockdown or knockout of *Aldh2* in ECs markedly increases the expression of EC barrier markers associated with focal adhesions and tight junctions, thereby restoring endothelial integrity and suppressing early aortic dilation in abdominal aortic aneurysm models at 7 and 14 days after Ang II infusion [104]. Metabolic reprogramming, such as enhanced glycolysis, can exacerbate local microenvironmental disturbances, where lactate accumulation induces increased acidity that activates matrix metalloproteinases (MMPs), accelerates extracellular matrix degradation, and ultimately weakens the mechanical stability of the vascular wall [75]. Single-cell sequencing has revealed the markedly increased expression of important glycolytic enzymes such as HK2 and PFKFB3 in ECs within lesion regions, accompanied by a proinflammatory and procoagulant phenotypic shift along with mitochondrial malfunction [76]. Moreover, impaired endothelial energy metabolism and consequent ATP depletion can disrupt intercellular integrity and increase vascular permeability [105]. Endothelial metabolic disorders resulting from hyperhomocysteinemia compromise barrier integrity and promote aneurysm progression [77]. The mitochondrial dysfunction–associated accumulation of acetyl-CoA can further modulate histone acetylation, thereby influencing inflammatory gene expression and amplifying vascular wall inflammation [89]. Endothelial-derived microvesicles can carry metabolic enzymes or miRNAs, which modulate smooth muscle cell function via paracrine signaling and participate in aortic dilation, although the underlying mechanisms are incompletely understood [106].

**Fig. 6.**
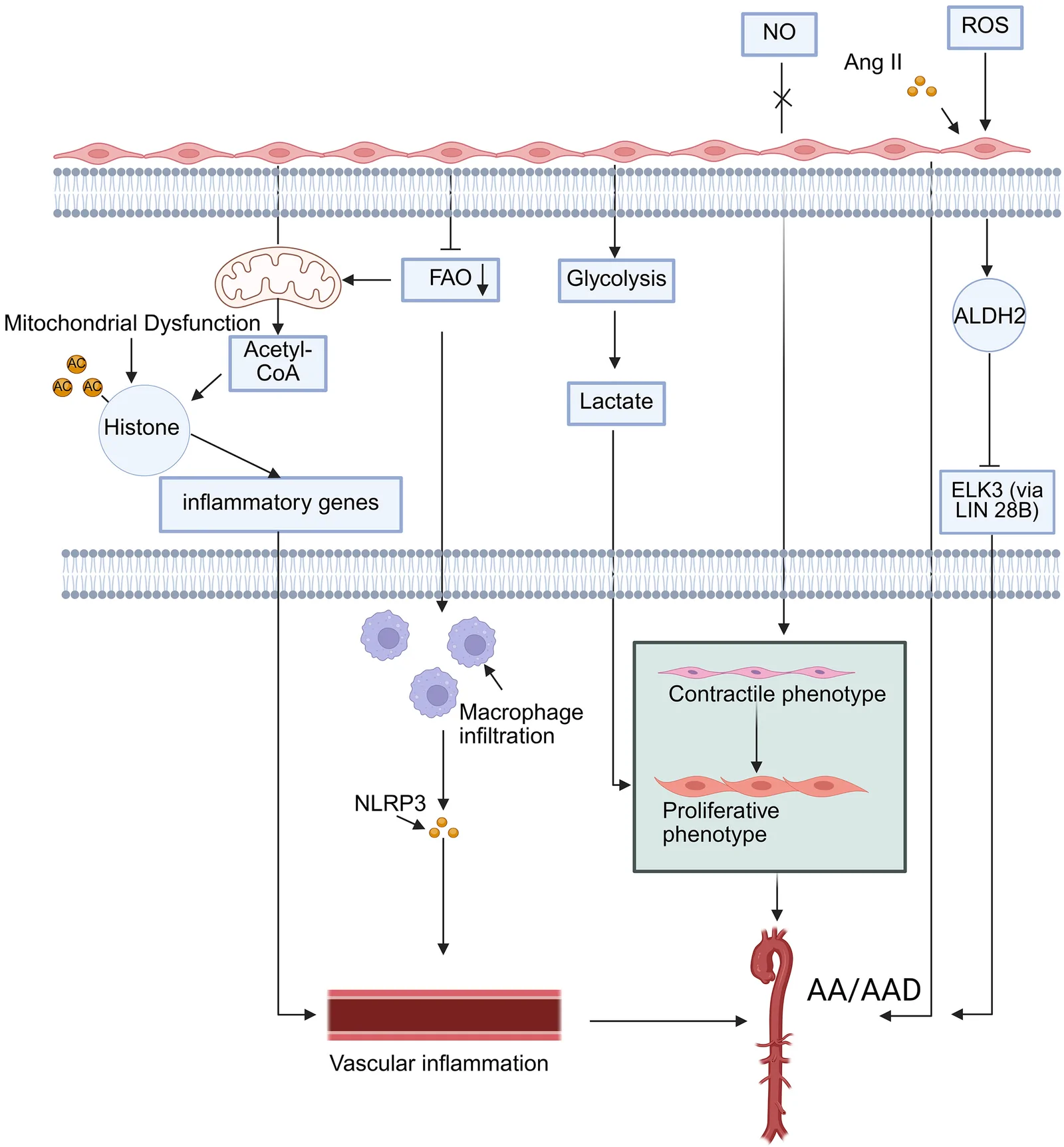
**Metabolic and inflammatory mechanisms involved in aortic aneurysms and aortic dissections**. Mitochondrial dysfunction and disturbed fatty acid oxidation contribute to inflammatory activation and macrophage infiltration. Enhanced glycolysis and lactate accumulation are associated with extracellular matrix degradation and vascular wall injury. In parallel, oxidative stress and altered ALDH2–ELK3 signaling are involved in endothelial dysfunction and vascular remodeling. These metabolic, oxidative, and inflammatory alterations collectively promote vascular inflammation, extracellular matrix disruption, and the progression of aortic aneurysms and aortic dissections. Created in BioRender.

These findings suggest that endothelial metabolic dysregulation extends beyond local effects, influencing the metabolic characteristics of adjacent cells through intercellular signaling and creating a positive feedback loop that perpetuates degenerative changes in the vascular wall.

## 4. Therapeutic Strategies Targeting Endothelial Metabolic Reprogramming in CVDs

### 4.1 Natural Compounds

Natural compounds such as resveratrol, quercetin, and curcumin preserve EC functions by reducing oxidative stress and inflammation and promoting the synthesis of NO and other substances. Resveratrol alleviates endothelial injury by suppressing PI3K/AKT-associated glycolytic activation, as evidenced by reduced PFKFB3, GLUT1, HK2, glucose uptake, and lactate production. In parallel, studies in other cellular contexts suggest that resveratrol can modulate the ERK/PKM2 axis, while ERK-dependent PKM2 nuclear translocation is known to promote glycolytic reprogramming [107,108]. Clinical studies have shown that quercetin supplementation may reduce blood pressure, whereas its protective effects against myocardial hypertrophy have been demonstrated primarily in preclinical studies; however, the underlying mechanisms remain incompletely understood [109]. Curcumin can increase high-density lipoprotein cholesterol levels, reduce low-density lipoprotein cholesterol levels, and markedly improve the endothelial function in postmenopausal women [110].

### 4.2 Traditional Chinese Medicine Formulations

Qili Qiangxin capsules improve the utilization and metabolism of glucose, increase ATP production, and alleviate ischemic injury in microvascular cells by upregulating HIF-1α, GLUT1, HK2, PKM2, PFK1, and LDHA [111]. Tongxinluo capsules protect ECs by inhibiting the free fatty acid–induced p38-MAPK signaling pathway and alleviating symptoms such as coronary AS [112]. Danshen polyphenolic acids reduce aortic EC injury in mice on a high-cholesterol diet [113]. Plasma metabolomics studies on rats with microcirculatory dysfunction identified several species linked to the metabolism of linoleic acid, glutathione, pantothenate and CoA biosynthesis, pentose and glucuronate interconversion, pyruvate metabolism, and the citric acid cycle. These findings suggest that Danshen polyphenolic acids can protect ECs by modulating endothelial metabolic pathways, although the precise mechanisms require further investigation [114]. Compound Danshen dripping pills are one of the most often used traditional Chinese medicine formulations that also show clinical efficacy. In particular, Danshen polyphenolic acids delay disease progression by hindering endothelial apoptosis and mitochondrial autophagy [115]. Under high-glucose and high-lipid conditions, salvianolic acid B mitigates endothelial injury by downregulating Rho-associated coiled-coil containing protein kinase 1 (ROCK1) and inhibiting the expression of mitochondrial fission proteins, including phosphorylated dynamin-related protein 1 (DRP1) and mitochondrial fission 1 protein (FIS1) [116].

### 4.3 Synthetic Drugs and Novel Formulations

Metformin, a classic hypoglycemic agent, inhibits NF-κB activation via the IKK/IKBα/NF-κB and PI3K-Akt signaling pathways. In diabetes, a process associated with eNOS results in the generation of the superoxide anion instead of NO, a phenomenon known as eNOS uncoupling. The decrease in the levels of BH4, an essential cofactor for eNOS, further contributes to this uncoupling. BH4 levels in diabetic mice treated with metformin were reported to notably exceed those in the control group, and the former group also featured a marked reduction in EC apoptosis and oxidative stress [117]. Metformin can modulate fatty acid metabolism in ECs via fatty acid binding protein 4 (FABP4), reducing lipid accumulation and inflammatory responses induced by saturated fatty acids, and participates in the transfer of hexose and reverses endoplasmic reticulum stress in ECs [118]. Sodium-glucose co-transporter 2 (SGLT2) inhibitors, such as dapagliflozin (DAPA), target endothelial metabolism and improve cardiovascular outcomes [119,120]. DAPA can inhibit the AKT/mTOR pathway, thereby enhancing autophagy in ECs and alleviating the lipotoxicity induced by low-density lipoprotein [121]. In myocardial ischemia–reperfusion injury, DAPA inhibits the cofilin-dependent xanthine oxidase (XO)-sarcoplasmic/endoplasmic reticulum Ca^2+^ ATPase 2a (SERCA2)-calcium/calmodulin-dependent protein kinase II (CaMKII) pathway, preventing F-actin depolymerization and cytoskeletal degradation and notably attenuating hypoxia/reoxygenation-induced apoptosis [122]. Furthermore, GLP-1 receptor agonists, such as semaglutide, are being investigated for their potential to improve endothelial function and reduce vascular inflammation [123]. Experimental compound I-152 has been shown to enhance GSH production and reduce oxidative stress and ROS levels in hypoxic ECs, thereby protecting endothelial function and alleviating vascular injury [90].

## 5. Conclusion and Perspectives

ECs are increasingly recognized as metabolically plastic regulatory units actively contributing to CVD initiation and progression. Under pathological stimuli, including hyperglycemia, dyslipidemia, hypoxia, disturbed shear stress, and chronic inflammation, ECs experience a coordinated remodeling of glucose, lipid, and amino acid metabolism, which causes NO deficiency, mitochondrial dysfunction, oxidative stress, inflammatory activation, barrier disruption, impaired angiogenesis, and vascular remodeling. These observations indicate that endothelial metabolic reprogramming is not merely a secondary response to vascular injury but may serve as a central mechanistic link between metabolic stress and cardiovascular dysfunction.

Future studies should focus on several unresolved issues. First, disease-driving metabolic alterations should be distinguished from adaptive or compensatory metabolic responses in ECs. Second, endothelial metabolic remodeling should be characterized in a vascular-bed-, disease-stage-, and cell-subtype-specific manner using single-cell omics, spatial metabolomics, isotope tracing, and lineage-tracing approaches. Third, the causal roles of key metabolic intermediates, including lactate, acetyl-CoA, α-KG, succinate, NADPH, Glu, and SAM, should be systematically examined, particularly in relation to endothelial epigenetic remodeling, inflammatory transcriptional programs, redox regulation, and intercellular communication within the vascular microenvironment.

Therapeutically, future work should prioritize endothelial-selective metabolic modulation rather than broad systemic metabolic intervention. Targeted delivery systems such as nanoparticles, engineered extracellular vesicles, antibody-guided platforms, and vascular-bed-specific carriers may improve therapeutic precision while minimizing off-target metabolic effects. In parallel, the integration of multiomics datasets with artificial intelligence may enable patient stratification, the identification of disease-stage-specific metabolic vulnerabilities, and the prediction of therapeutic responses. These efforts will provide a stronger mechanistic and translational foundation for developing metabolism-based precision therapies for CVDs.
